# Low salt and low calorie diet does not reduce more body fat than same calorie diet: a randomized controlled study

**DOI:** 10.18632/oncotarget.23959

**Published:** 2018-01-04

**Authors:** Hye Jin Kang, Dae Won Jun, Seung Min Lee, Eun Chul Jang, Yong Kyun Cho

**Affiliations:** ^1^ Department of Internal Medicine, Hanyang University College of Medicine, Seoul, South Korea; ^2^ Department of Food and Nutrition, Sungshin Women's University, Seoul, South Korea; ^3^ Department of Occupational and Environmental Medicine, Soonchunhyang University College of Medicine, Cheonan, South Korea; ^4^ Department of Internal Medicine, Kangbuk Samsung Hospital, Sungkyunkwan University School of Medicine, Seoul, South Korea

**Keywords:** low salt, obesity, diet

## Abstract

**Background:**

Recent several observational studies have reported that high salt intake is associated with obesity. But it is unclear whether salt intake itself induce obesity or low salt diet can reduce body fat mass. We investigated whether a low salt diet can reduce body weight and fat amount.

**Matrials and Methods:**

The randomized, open-label pilot trial was conducted at a single institution. A total of 85 obese people were enrolled. All participants were served meals three times a day, and provided either a low salt diet or control diet with same calorie. Visceral fat was measured with abdominal computer tomography, while body fat mass and total body water was measured with bio-impedance.

**Results:**

Reductions in body weight (–6.3% vs. –5.0%, *p* = 0.05) and BMI (–6.6% vs. –5.1%, *p* = 0.03) were greater in the low salt group than in the control group. Extracellular water and total body water were significantly reduced in the low salt group compared to the control group. However, changes in body fat mass, visceral fat area, and skeletal muscle mass did not differ between the two groups. Changes in lipid profile, fasting glucose, and HOMA-IR did not differ between the two groups.

**Conclusions:**

A two-month low salt diet was accompanied by reduction of body mass index. However, the observed decrease of body weight was caused by reduction of total body water, not by reduction of body fat mass or visceral fat mass.

## INTRODUCTION

Recently, multiple studies have shown a strong association between a high salt diet and obesity after adjusting total calorie intake [1, 2]. A study of 86 Swedish men found that high salt intake had a positive relation with body weight and body mass index (BMI), even after correcting for calories [3]. Another study that utilized the results of the Korea National Health and Nutrition Examination Survey (KNHANES) reported a significant association of salt intake with obesity and central obesity even after correcting for total energy intake [4]. The Danish MONICA study, a retrospective cohort study carried out in Denmark, reported an association between body fat mass and sodium consumption [2]. In a cross sectional study with 184 subjects in the UK, waist circumference and body fat mass increased with increased salt intake [5]. Most previous studies have reported that high salt intake is related to obesity, but all were either cohort studies or cross-sectional studies. Due to the limits of observational studies, these studies could not determine if there is a causal relationship between salt intake and obesity. Thus, it is unclear whether obesity is caused directly by salt intake or by bad eating habits accompanied by excessive sodium consumption. Moreover, no possible mechanism between high salt diet and obesity was discussed in any of these studies. It is not clear whether weight gain caused by a high salt diet is attributable to an increase in total body water or body fat mass. Two studies have tested if salt intake is related to body fat mass, but both are retrospective, observational studies that did not properly correct for various factors influencing fat mass other than the level of salt intake. On the contrary, no study on the association between a low salt diet and weight loss has been completed. For these reasons, a long-term, randomized, controlled study with a high level of evidence about how low salt intake has an impact on obesity is needed.

The objective of this study is to investigate the impact of low salt diet on body weight and body fat mass, furthermore to identify any adverse effects.

## RESULTS

### Basic characteristics

The characteristics of participants in the control diet and the low salt group are shown in Table 1. The majority were office workers or housewives by occupation. There was no difference between the control group and the low salt group in salty taste acuity prior to dietary intervention. Average levels of salt intake were 4,170 mg/day in the control group and 4,150 mg/day in the low salt group, which was not significantly different between the two groups. Baseline total calorie intake, obesity-related metabolic markers, and level of physical activity did not differ between the two groups.

**Table 1:** Baseline characteristics of study participants

	Control group (*n =* 43)	Low salt group (*n =* 41)	*p*-value^*^
Age (year)	42.3 ± 7.3	42.5 ± 7.6	0.881
Height (cm)	161.0 ± 6.8	161.4 ± 8.1	0.823
Body weight (kg)	73.9 ± 8.9	74.8 ± 12.7	0.700
Body mass index (kg/m^2^)	28.4 ± 2.4	28.6 ± 3.7	0.781
Waist circumference (cm)	96.9 ± 6.1	95.8 ± 7.7	0.467
Total abdominal fat area	394.7 ± 83.3	399.0 ± 112.7	0.845
Visceral fat area	143.8 ± 50.7	145.7 ± 56.9	0.872
Systolic blood pressure (mmHg)	128.4 ± 15.7	129.5 ± 15.1	0.762
Diastolic blood pressure (mmHg)	76.8 ± 10.1	74.9 ± 8.3	0.373
ALT (U/L)	25.2 ± 25.9	20.4 ± 10.4	0.278
AST (U/L)	21.8 ± 10.1	21.8 ± 6.3	0.979
γ-GGT (U/L)	45.9 ± 83.8	30.6 ± 30.0	0.280
Fasting glucose (mg/dl)	86.5 ± 16.6	82.8 ± 21.0	0.381
Triglyceride (mg/dl)	154.2 ± 88.1	154.0 ± 114.6	0.993
HDL-cholesterol (mg/dl)	50.0 ± 11.0	53.3 ± 11.9	0.208
Fasting insulin (μIU/m)	6.4 ± 3.6	6.7 ± 4.2	0.789
HOMA-IR	25.5 ± 18.5	25.7 ± 22.7	0.960
Skeletal muscle mass (kg)	43.2 ± 7.0	43.8 ± 9.1	0.142
Body fat mass (kg)	27.2 ± 5.8	27.8 ± 8.0	0.132
Intracellular water (ℓ)	20.8 ± 3.5	21.3 ± 4.1	0.288.
Extracellular water (ℓ)	12.8 ± 2.0	13.1 ± 2.6	0.096
Total body water (ℓ)	33.7 ± 5.4	34.5 ± 6.7	0.193
CT-scan Visceral fat area	143.8 ± 49.6	146.3 ± 56.3	0.942
CT-scan Intra-abdominal fat area	396.9 ± 84.4	397.1 ± 111.9	0.137

ALT: Alanine aminotransferase; AST: Aspartate aminotransferase; γ-GGT: γ-glutamyl trasnferase; TG: Triglyceride; HDL-cholesterol: High density lipoprotein cholesterol; HOMA-IR: Homeostatic model assessment - insulin resistance; CT-scan: Computer tomography scan (^*^p < 0.05 by Student *t* test between control and low salt group).

### Effects of low salt diet on weight and body composition

**Figure 1:**
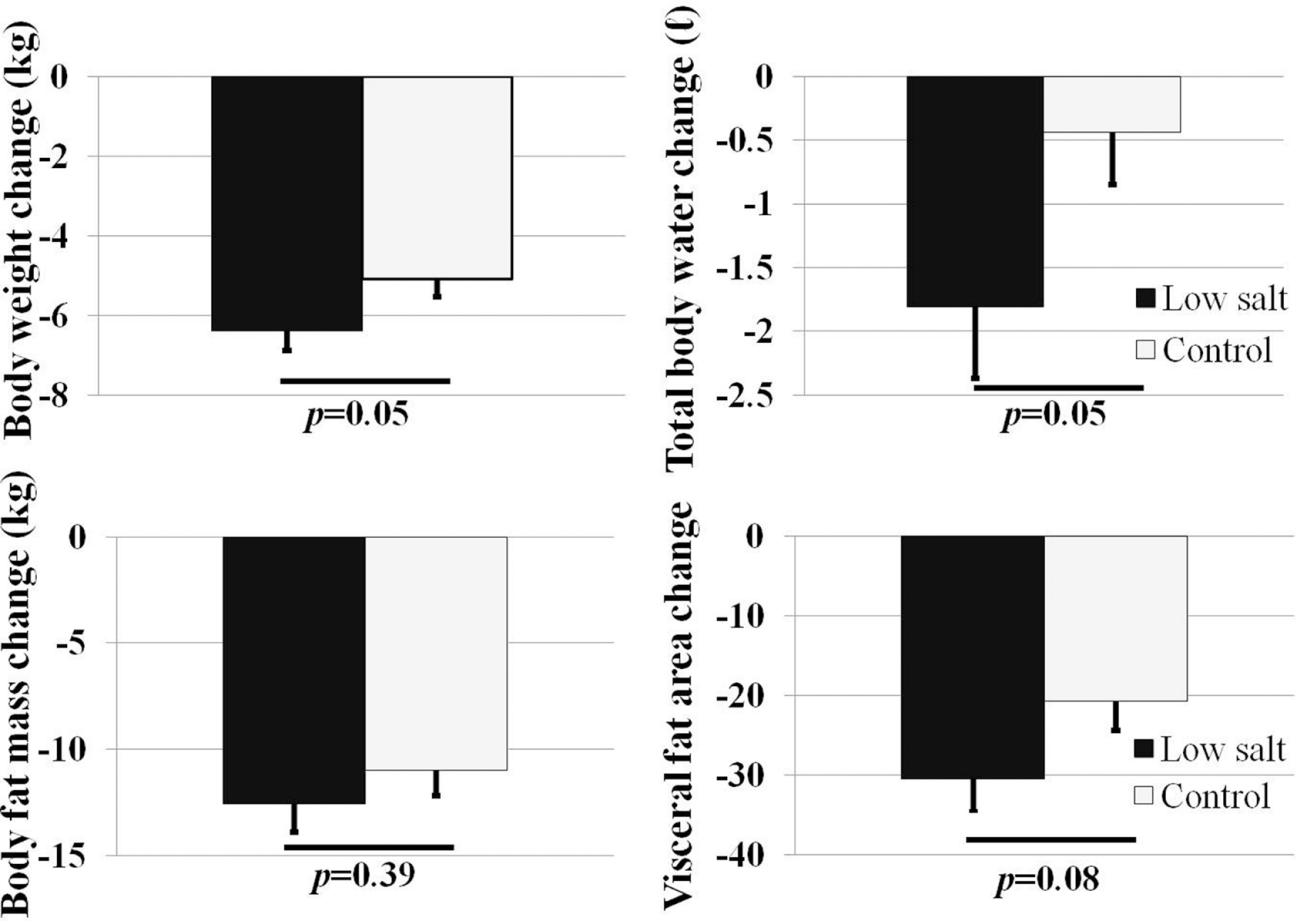
Comparison of total body weight, body composition in low salt and control group after 2 months (^*^ *p* < 0.05 by Student *t* test between control and low salt group)

The percent of body weight reduction (–6.3% vs. –5.0%, *p* = 0.05) and BMI reduction (–6.6% vs. –5.1%, *p* = 0.03) were greater in the low salt group than in the control group (Table 2, Figure 1). Total body water, body fat mass, and fat distribution were measured by bio-impedance. The percent of extracellular water change (–0.1kg vs. –1.5kg, *p* = 0.04) and total body water change (–0.4kg vs. –1.8kg, *p* = 0.05) were significantly greater in the low salt group compared to the control group. However, no difference in body fat mass loss or skeletal muscle mass variation was found between the two groups (Table 2, Figure 1). The impacts of salt intake on visceral fat were assessed using abdominal CT. The low salt group showed greater reduction in visceral fat area than the control group, but without statistical difference (–20.6 cm^2^ vs. –30.4 cm^2^, *p* = 0.08).

**Table 2:** Comparison of total body weight, body composition in control and low salt group after 2 months

	Control group			Low salt group			Change rate (%) compare to baseline		
	Baseline	2 month	^*^p	Baseline	2 month	^*^p	Control	Low salt	^**^p
Body weight change (kg)	74.1 ± 8.9	70.3 ± 8.7	< 0.001	75.1 ± 12.7	70.3 ± 12.6	< 0.001	**–5.0 ± 2.9**	**–6.3 ± 3.1**	**0.05**
**BMI change (kg/m^2^)**	28.6 ± 2.5	27.1 ± 2.5	< 0.001	28.6 ± 3.7	26.7 ± 3.8	< 0.001	**–5.1 ± 3.0**	**–6.6 ± 3.1**	**0.03**
Waist circumference (cm)	97.0 ± 6.4	89.6 ± 7.7	< 0.001	95.9 ± 7.6	88.0 ± 8.7	< 0.001	–7.4 ± 4.7	–7.9 ± 3.8	0.60
Skeletal muscle mass change (kg)	43.5 ± 7.0	43.2 ± 6.8	0.154	43.4 ± 8.9	42.9 ± 8.0	0.643	0.5 ± 2.6	0.1 ± 11.3	0.83
Body fat mass change (kg)	26.6 ± 5.5	23.5 ± 5.1	< 0.001	28.1 ± 8.0	24.5 ± 8.1	< 0.001	–11.0 ± 7.7	–12.6 ± 8.7	0.39
Intracellular water change (ℓ)	20.9 ± 3.5	20.8 ± 3.5	0.121	21.4 ± 4.1	20.7 ± 3.9	0.001	–0.6 ± 2.7	–2.0 ± 4.0	0.07
**Extracellular water change (ℓ)**	12.8 ± 2.0	12.8 ± 1.9	0.597	13.0 ± 2.5	12.7 ± 2.4	0.006	**–0.1 ± 2.9**	**–1.5 ± 3.1**	**0.04**
**Total body water change (ℓ)**	33.9 ± 5.4	33.7 ± 5.3	0.217	34.2 ± 6.5	33.7 ± 6.3	0.001	**–0.4 ± 2.7**	**–1.8 ± 3.6**	**0.05**
CT-scan Visceral fat area (cm^2^)	143.8 ± 50.7	121.1 ± 40.4	0.872	145.7 ± 56.9	115.7 ± 43.2	0.573	–20.6 ± 23.2	–30.5 ± 26.0	0.08
CT-scan Intra-abdominal fat area (cm^2^)	394.7 ± 83.3	352.2 ± 86.7	0.845	399.0 ± 112.7	344.9 ± 117.2	0.758	–44.3 ± 33.6	–53.4 ± 46.2	0.32

BMI: body mass index; CT-scan: Computer tomography scan (^*^p < 0.05 by paired *t* test, ^**^p < 0.05 by Student *t* test).

### Effects of a low salt diet on metabolic parameters

Compared to values prior to treatment, ALT, AST, GGT, triglycerides, fasting glucose, and HOMA-IR were significantly decreased in both groups at the second month of dietary intervention (Table 3). There was no difference between the two groups in absolute values of ALT, AST, GGT, triglycerides, fasting glucose, or HOMA-IR at the second month. Systolic and diastolic pressure decreased in both control and low salt group during two months (Table 3). Systolic blood pressure reduction was greater in the low-salt group, but there was no statistically significant difference between the two groups.

**Table 3:** Comparison of metabolic parameter change in control and low salt group

	Control group			Low salt group			Mean difference		
	Baseline	2 month	^*^p	Baseline	2 month	^*^p	Control	Low salt	^**^p
ALT (U/L)	25.5 ± 25.7	14.5 ± 11.5	< 0.001	19.9 ± 9.8	11.5 ± 4.7	< 0.001	–11.0 ± 18.4	–8.5 ± 8.4	0.425
AST (U/L)	22.1 ± 10.2	14.2 ± 6.5	< 0.001	21.3 ± 5.7	12.8 ± 2.9	< 0.001	–7.9 ± 7.9	–8.6 ± 5.4	0.647
γ-GGT (U/L)	44.6 ± 81.9	25.8 ± 45.5	0.004	27.8 ± 19.8	15.3 ± 10.2	< 0.001	–18.8 ± 39.9	–12.5 ± 12.2	0.341
Triglyceride (mg/dl)	156.3 ± 86.0	97.5 ± 52.5	< 0.001	159.2 ± 121.0	84.2 ± 57.4	< 0.001	–58.8 ± 84.7	–75.0 ± 96.7	0.422
HDL-cholesterol (mg/dl)	49.7 ± 11.3	36.2 ± 12.2	< 0.001	52.8 ± 12.1	35.6 ± 7.6	< 0.001	–13.5 ± 7.6	–17.3 ± 10.0	0.060
Fasting insulin (μIU/m)	8.36 ± 6.6	8.25 ± 7.5	0.822	7.21 ± 4.0	6.20 ± 3.3	0.102	–0.1 ± 3.0	–1.0 ± 3.5	0.263
Fasting glucose (mg/dl)	86.7 ± 16.1	70.5 ± 11.8	< 0.001	82.7 ± 20.9	68.4 ± 12.5	< 0.001	–16.2 ± 16.2	–14.4 ± 17.9	0.622
HOMA-IR	33.4 ± 28.2	26.2 ± 27.2	0.004	28.3 ± 23.3	19.6 ± 12.8	0.012	–6.8 ± 12.7	–8.7 ± 19.0	0.634

BMI: body mass index; CT-scan: Computer tomography scan; HU: Hounsfield Unit; ALT; Alanine aminotransferase; AST; Aspartate aminotransferase; γ-GGT; γ-glutamyl transferase; HDL-cholesterol: High density lipoprotein cholesterol; HOMA-IR: Homeostatic model assessment - insulin resistance (^*^p <0.05 by paired *t* test, ^**^p <0.05 by Student *t* test).

### Change in salty taste acuity

Baseline salty taste acuity was the same in both groups at the start of the study. At the second month of dietary intervention, the control group showed no change in salty taste acuity, while the salty taste acuity of the low salt group was decreased.

### Compliance with study diets

There was no difference in the level of total calorie consumption between the control group (1,479 kcal) and the low salt group (1,501 kcal) prior to dietary intervention. During the 2-month intervention period, average total calorie consumption did not differ between the control group (1,314 kcal) and the low salt group (1,287 kcal) (*p* = 0.74).

There was no difference in meal consumption between the two groups; participants in the control group consumed an average of 80.0% of the three meal boxes each day, while those in the low salt group consumed 78.0%. Participants recorded in food diaries any additional snacks consumed other than those provided in meal boxes. The average calories consumed from non-meal box snacks was 243.2 kcal in the control group and 227.9 kcal in the low salt group, which was not statistically different. The amount of sodium consumed from additional snacks was 273.1 mg and 237.1 mg in the control and low salt groups, respectively, representing no statistical differences. Compliance during the dietary intervention period was evaluated using a 3-day food diary and 24-hour urine sodium excretion (Table 4). The levels of salt intake identified in the 3-day food diary were 4,170 mg and 4,160 mg in the control group and the low salt group, respectively, demonstrating no difference between the two groups (*p* = 0.96). However, levels of salt intake after the 2-month dietary intervention were 3,500 mg in the control group and 1,720 mg in the low salt group (*p* < 0.001). When sodium intake was assessed through 24-hour urine collection during the second month of dietary intervention, sodium excretion was significantly lower in the low salt group compared to that in the control group.

**Table 4:** Estimated sodium intake according to survey method

	Control diet (5 gram/day)			Low salt diet (2 gam/day)		
	Baseline	2 month	p^*^	Baseline	2 month	p^*^
Total food intake (g)	1488.1 ± 604.2	1314.9 ± 362.3	0.097	1510.6 ± 571	1289.2 ± 364	0.021
Total energy intake (kcal)	1971.6 ± 506.8	1445.5 ± 336.2	< 0.001	1986.6 ± 690	1325.4 ± 219	< 0.001
Na intake, 3-day diary (g/day)	4168.9 ± 1885.0	3508.4 ± 876.2	0.220	4150.1 ± 1734.3	1725.9±502.1	< 0.001
24-HU Na excretion (mEq/day)	172.5 ± 64.5	152.4 ± 67.6	0.056	186.9 ± 78.0	123.4 ± 49.6	0.001

Na: sodium; 24-HU Na excretion: 24-hours urine sodium excretion (^*^p <0.05 by Student *t* test between control and low salt group).

## DISCUSSION

A two-month low salt diet decreased body weight, and the percent of body weight reduction was greater than that in the control diet group. The decrease in body weight and BMI were caused by reduction in fluid retention, not by reducing body fat mass. To the best of our knowledge, this is the first randomized controlled study testing whether salt restriction decreases body weight or body fat mass.

To date, 17 studies on the relationship between salt intake and obesity have been published, including two cohort studies, [2, 6] 14 cross sectional studies, [1, 4, 5, 7–17] and one case-control study [18]. Although most previous studies have suggested an association between salt intake and obesity, no studies have clearly demonstrated an association between salt intake and fat mass. However, it is still unclear whether salt intake can increase body fat mass or trigger obesity.

Two previous studies have investigated the relationship between salt intake and fat mass [2, 5]. In the Danish MONICA study, a retrospective cohort study carried out in Denmark, 10-year follow-up of 215 subjects showed that body fat mass increased with an increase in sodium consumption, while free fat mass decreased [2]. The Danish MONICA study also analyzed the correlation between salt intake and body fat mass over 10 years. However, the amount of salt intake was evaluated only once at baseline, and there was no follow up survey regarding changing diet behavior after that. In addition, only 215 of 600 participants (35.8%) could be followed up. More critically, there was no adjustment of energy intake or soft drink intake. A second study was performed in the UK [5]. In a cross-sectional study of 184 subjects, waist circumference and body fat mass increased with increased salt intake, which was measured through 24-hour urinary sodium excretion, while lean body mass decreased. In the UK study, the 24-hour urinary sodium excretion and anthropometric data were not recorded simultaneously and were tested at different points. Moreover, body fat mass and lean body mass were not directly measured through bio-impedance or dual energy X-ray absorptiometry. Body fat mass was calculated using a double-labeled water method formula. Above all, no causal relationship was found since both studies were observational in design. This study is the first randomized controlled trial examining the effects of a low salt diet on body weight and body fat mass using a controlled diet for 2 months.

Other unsolved issue regarding low salt diet is concern over potential adverse effects that have been suggested by some studies [19, 20]. There are some concerns about deteriorating metabolic parameters due to low salt intake. Several studies have reported that low salt diet can increase insulin resistance and levels of total cholesterol, low density lipoprotein cholesterol, and triglycerides [8, 18]. However, none of these studies were carried out for longer than two weeks, and some studies were performed using an extremely low salt diet (< 1 g sodium per day) with a small number of subjects. The influence of low sodium diet on insulin resistance and dyslipidemia is unclear and has shown variable results [21, 22]. A total of 25 studies related to salt intake and insulin resistance have been published by 2015 and have reported different results [23]. Nine of these studies reported that insulin resistance would increase, while seven reported that it would decrease; other studies concluded that no significant differences were observed. Studies that reported negative effects of restricted salt intake on insulin resistance have suggested that restrictions in sodium consumption decrease fluid retention in the body; to compensate for this loss, there are increases in the amounts of epinephrine, renin, and angiotensin, which lead to insulin resistance because these hormones restrict insulin function [18]. However, most studies performed with extremely low salt diets (< 1 g sodium per day) have been conducted over a very short period (< 2 weeks). While these studies, which provided frozen meal as an intervention, show a clearer causal relationship than would a cross sectional study, it is difficult to conclude that low salt intake has a long-term effect because the intervention period is too short [24, 25]. Our study did not find a significant effect on insulin resistance and lipid metabolism from consuming a low salt diet of 2 grams of sodium daily for 2 months.

There were some limitations in this study. First, while this study was designed as a randomized controlled trial, double-blinding was not possible due to the nature of the low salt diets that were provided. However, the primary endpoint is body weight while the secondary endpoints are body composition, drawn from bioelectric impedance, and biochemical markers. Objective parameters were used as the primary or secondary endpoints. Secondly, while the three-day diet diary and the 24-hour urinary sodium excretion were evaluated to make sure the amount of sodium consumption, it still may not perfect to reflect the actual amount of salt intake. Thirdly, although this is the longest clinical trial testing a low salt diet, a two-month time period may not be sufficient to fully assess metabolic changes and obesity-related parameters. Despite the absence of statistical significance, a significant decrease in body fat mass and visceral fat amount was notable. Intervention study of a longer period would be required to determine the causal relationships between salt intake and fat amounts.

This study demonstrated that BMI and body weight were decreased after a two-month low salt diet program, without significantly adverse effects. However, a low salt diet did not reduce body fat mass or visceral fat area. A two-month low salt diet was accompanied by reduction of body mass index which was caused by reduction of total body water, not by reduction of body fat mass or visceral fat mass.

## MATERIALS AND METHODS

### Design

This study was a randomized, open label, parallel, pilot trial designed to examine the efficacy of a low salt diet versus control diet for two months. The study was approved by the institutional review board of Hanyang University Hosptial. This study was registered with the Clinical Research Information Service of the Korea Centers for Disease Control and Prevention prior to the commencement of research (KCT0001084, https://cris.nih.go.kr).

### Participants

Subjects were adults aged 19 to 70 years, all of whom participated voluntarily. This study was conducted at a single institution, Hanyang University Medical Center. The study populations for this study were white-collar workers from a single center and housewives, and data was stratified by sex and BMI. All participants were measured for height and weight and were eligible for the study if they were “obese.” The definition of obesity used in Asian countries is different from that used in Western countries. In this study, participants with a body mass index (BMI) of 25kg/m^2^ or more were considered “obese” [26, 27]. All participants provided written informed consent.

Exclusion criteria was such as following. Participants who were newly diagnosed with hypertension, and dyslipidemia in the last six months. Subjects who diagnosed with diabetes mellitus-diabetes mellitus was defined as fasting glucose ≥ 126 mg/dL (7.0 mmol/L) or hemoglobin A1C ≥ 6.5% (48 mmol/mol) or those who took oral hypoglycemic agents. Those who take diuretics included thiazides. Those who receive consultation on diet and nutrition in the last six months. Those diagnosed with a malignancy in the previous year. Those who received stomach surgery. Those who underwent thyroidectomy in the last three years, those who regularly visit the hospital more than four times a year and take drugs due to liver, heart, or kidney disease. Men consuming 210 g of alcohol per week or women consuming 140 g of alcohol per week; [28, 29] and those working night shifts were excluded.

### Randomization and allocation

Subjects were randomly allocated to either the low salt or control group in a 1:1 ratio by computer. We used stratified randomization method according to sex and BMI. Because the taste of food in the low salt diet was flat, participants easily noticed it. Thus, blinding and allocation concealment were not maintained. This study was performed as an open label study.

### Follow-up

**Figure 2:**
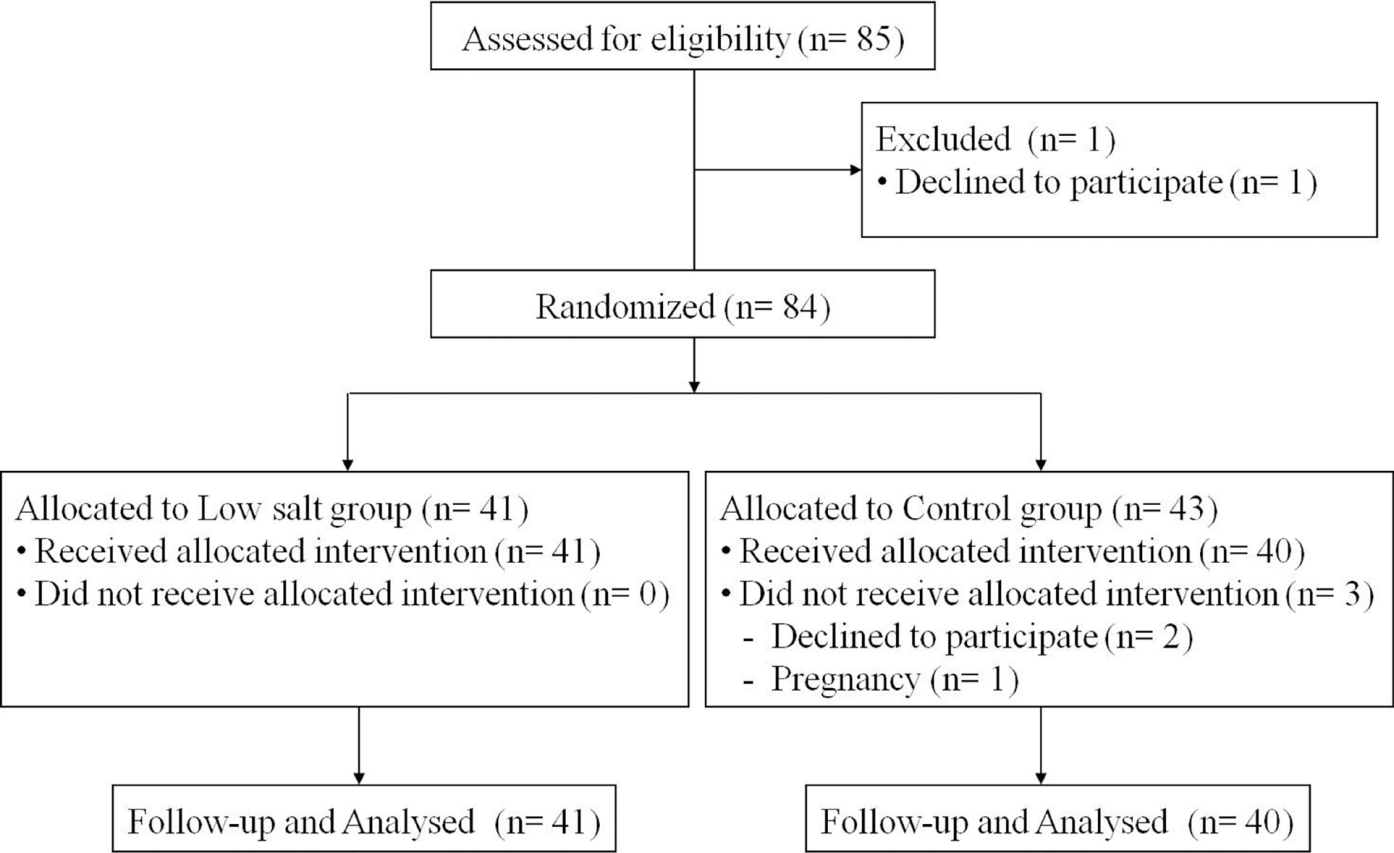
Consort flow chat of randomized controlled study of low salt and control diet

A total of 85 subjects were enrolled this study and followed up from April 2014 to May 2014 (Figure 2). One participant withdrew consent during the screening process, and a total of 84 subjects were randomly assigned to two groups: 41 in the low salt group and 43 in the control group. For two months when a meal box was offered three meals a day, three participants were eliminated from the control group: one participant became unexpectedly pregnant and two withdrew consent at the 3rd or 4th week of diet intervention due to ‘skimpy meals’ based on calorie restriction. Participants were asked to write a food diary every day about the meals provided, and nutritionists reviewed these diaries every week. Those who continued to consume more than 500 kcal in addition to the meals provided or who ate less than 60% of the meal provided were also eliminated from the study. Sample size was calculated with sample size equation. We used the weight loss values using previous study that assessed weight loss in patients for low salt diet. For a significance level of α = 0.05, a statistical power of 80%, and a substantial difference in weight loss of 1.78 kg (experimental arm; –4.42 kg vs. control arm; –2.64 kg). Drop-out rate estimated 10%. Final target population was decided as 90 subjects (KCT0001084, https://cris.nih.go.kr).

### Dietary intervention

All participants were served meals three times a day, five days a week from Monday through Friday for two months. Lunch and dinner were provided to each participant, and breakfast was included in a meal box with dinner. During the weekend, the kinds and amount of foods consumed were recorded using a food diary. Low-calorie meals (an average 1600 kcal based on 25 kcal of body weight per day) were provided to all participants in order to promote weight loss [30]. The calorie and nutrient content of each meal were composed according to criteria recommended by the Dietary Reference Intakes of Koreans (fat 15–20%, protein 20%, and carbohydrate 60–65%) [31]. The same energy ratio was supplied to meals for both the low salt group and the control group.

The low salt group was served meals containing 2.0 gram of sodium per day, which is the World Health Organization (WHO)-recommended daily sodium intake, while the control group was served meals containing 5.0 gram of sodium per day [32]. The control meals were based on results from the 2009-2012 Korea National Health and Nutrition Examination Survey (KNHANES), which reported average sodium intakes of 4.5 and 6-6.5 gram per day for women and men, respectively. Among all participants, the average age was 42 years, and women accounted for 77%. The sex ratio, age, and calories supplied were corrected to set 5 gram of sodium for control diet, and the density of sodium ingested is similar to that of real life.

### Outcomes

The primary endpoint was change in percent body mass index compared to baseline. The secondary endpoints were percent of body weight reduction, fat amount, skeletal muscle mass, visceral fat area, and total body water compared to baseline.

### Measurement of clinical parameters

Weight and height were measured using an automatic extensometer. Quality control of the automatic extensometer was performed every six months. Waist circumference was measured by placing a tape measure around waist 2 cm above the highest point of the iliac crests while exhaling, [33] The waist was measured three times and recorded to the 0.1 centimeter without including the thickness of clothing.

Blood collection for biochemical markers was performed after 8-hour fasting. Lipid profile and biochemical markers including triglyceride (TG), high density cholesterol (HDL), serum glucose, insulin, alanine aminotransferase (ALT), aspartate aminotransferase (AST), and γ-glutamyl transferase (γ-GGT) levels were measured using an autoanalyzer (Olympus GmbH, Hamburg, Germany). Insulin resistance was calculated by the following formula: HOMA-IR = fasting insulin (μU/mL) × fasting plasma glucose (mmol/l)/22.5 [34]. Quality control of biochemical examinations was conducted once a year following the guidelines for external quality control of the Korean Association of Quality Assurance for Clinical Pathology.

The measurement of body composition was conducted using bio-impedance (INBODY 520 Body Composition Analysis). The parameters measured were body composition (body water, protein, minerals, and body fat), skeletal muscle-fat (weight, skeletal muscle mass, and body fat mass), obesity (BMI, body fat ratio, and abdominal fat ratio), and body balance (right arm, left arm, torso, right leg, and left leg). Visceral fat was analyzed using abdominal computer tomography(CT) scan, and the abdominal CT scan used in this study was multi-detector computer tomography with more than 16 slices [35]. Attenuation correction was performed on a daily basis. Abdominal CT scans were performed using a low-dose technique that minimizes radiation exposure (120 kVp, 50-75 mAs), and the slice thickness was 5 mm.

### Lifestyle surveys

Intensity, times, and duration of exercise were evaluated to survey daily life habits using a standard questionnaire used by the KNHANES [36]. Intensity of exercise defined as follows. Severe physical activity defined such as running (jogging), mountain climbing, fast bike riding, fast swimming, soccer, basketball, skipping rope, squash, playing singles tennis, and carrying heavy objects. Moderate physical activity defined such as slow swimming, playing doubles tennis, volleyball, badminton, table tennis, carrying light objects, and walking. Smoking, smoking days, smoking amount, drinking days, drinking amount, dietary supplement intake, and the kinds of dietary supplements were surveyed using a questionnaire used by the KNHANES.

A salty taste acuity test was completed using a computer program for salty taste assessment developed by the Department of Food and Drug Safety [37]. This method was designed to determine the favorite taste of the participant by providing water of five different salinity levels (bland, slightly bland, moderate, slightly salty, and salty). Participants who choose higher salinity water more strongly prefer a salty taste than those who choose lower salinity water.

### Assessment of compliance

Compliance of subjects was assessed by multifaceted approach. At first, we checked diet compliance with daily food diary during two months. All participant recorded food diary. Participants check intake rate of served meals as well as additional intake of snack during the weekday. Participants recoded the amount of rice and side dishes consumed every day in their served meal during the dietary intervention. Consumption of side dish and rice were scored as follows: 1 point if no side dishes or rice were consumed, 2 points if 25% of meals included side dishes or rice, 3 points if 50%, 4 points if 75%, and 5 points if all meals contained side dish and rice food. And during the weekend, the kinds and amount of foods consumed were recorded using semi-quantitative method. Nutritionists reviewed it, and also educated suggesting recipe during weekend. Second, three day food diary was investigated at the beginning and end of intervention. Food consumption of non-continuous three days (including one day of the weekend) were recorded prior to and post dietary intervention. The three-day food diary was used as material according to the CAN-Pro4.0 (Computer Aided Nutritional analysis program) developed by the Korean Nutrition Society. Two skilled nutritionists taught both the low salt group and the control group how to write a food diary using sample food models. A food diary was written by recalling food consumption during the past 24 hours. Third, 24-hour urine collection was performed at the beginning and end of intervention to assess consumption of salt intake [38, 39]. For the 24-hour urine test, urine from the second urination on the previous morning to the first urination on the following day was collected in an embalmed container. After measuring the gross volume of urine, urine creatinine concentration was determined to verify if an appropriate amount of urine was collected. Urine with creatinine level below 600 mg or over 3,200 mg was excluded.

Participant compliance was 80% on average, and those with compliance below 60% were considered withdrawn. There were two non-compliance participants in our study who withdrew consent on the account of insufficient calories intakes, and the change in the result is insignificant.

### Statistical analysis

We included all subjects from the intent-to-treat population, defined as all randomly assigned participants who received meal. Student’s *t*-test and Chi-square test were carried out to analyze salt intake-related indicators of body weight, BMI, body fat mass, and biochemical indicators. Paired *t*-test was also used to analyze indicators of body weight, BMI, body fat mass, and biochemical indicators. The statistics program used in this study was SPSS version 21 (IBM SPSS Inc., Chicago, IL, USA).
